# Comparison of histomorphometric and radiographic effects of growth guidance with tension-band devices (eight-Plate and FlexTack) in a pig model

**DOI:** 10.1080/17453674.2021.1873603

**Published:** 2021-01-19

**Authors:** Julia Sattelberger, Hauke Hillebrand, Georg Gosheger, Andrea Laufer, Adrien Frommer, Sebastian Appelbaum, Ahmed Abdul-Hussein Abood, Martin Gottliebsen, Ole Rahbek, Bjarne Moller-Madsen, Robert Roedl, Bjoern Vogt

**Affiliations:** a Department of Pediatric Orthopaedics, Deformity Reconstruction and Foot Surgery, University Hospital Muenster, Germany;; b General Orthopaedics and Tumour Orthopaedics, University Hospital of Muenster, Germany;; c Department of Research Methodology and Statistics in Psychology, University of Witten/Herdecke, Germany;; d Danish Paediatric Orthopaedic Research, University Hospital Aarhus, Denmark

## Abstract

Background and purpose — Temporary hemiepiphysiodesis for growth modulation in skeletally immature patients is a long-known technique. Recently the use of tension-band devices has become popular. This study compares 2 tension-band implants (eight-Plate and FlexTack) regarding their effects on the growth plate.

Animals and methods — 12 pigs in 2 equally sized groups (A and B) were investigated. The right proximal medial tibia was treated with either eight-Plate or FlexTack. The left tibia of the same pig was treated with the opposite implant. After 9 weeks all implants were removed. Animals in group B were then hosted for another 5 weeks. Histomorphometric analysis of the growth plate was carried out after 9 and 14 weeks, respectively. Radiographs were taken at implantation, removal, and after 14 weeks.

Results — Both tension-band devices achieved a statistically significant and clinically relevant growth inhibition, whereas the effect appeared to be more distinct after the use of FlexTack. Implant-related complications or physeal damage was not observed. After implant removal, rebound phenomenon was radiologically observed in all cases. The growth plates treated with eight-Plate showed a paradox reversal of the zonal distributions, with an increase of the proliferative zones at the previously arrested medial aspect of the physis and a decrease laterally.

Interpretation — Both eight-Plate and FlexTack proved to be appropriate devices for growth-guiding treatment. The radiographic evaluation showed a change in angular axes after treatment with each implant, while the correction appeared to be faster with FlexTack. The paradox cartilaginous reaction observed after removal of the eight-Plate might be a histopathological correlate for rebound phenomenon.

To achieve realignment of angular deformities in skeletally immature patients, remaining bone growth can be used for growth modulation procedures to avoid extensive surgical interventions (Stevens 2007). Temporary hemiepiphysiodesis (THE) aims to mechanically inhibit growth on one side of the physis through a bridging implant. The procedure has to be performed before skeletal maturity, to maintain sufficient potential for correction while also reducing the risk of relapse of the deformity after implant removal (rebound phenomenon). Blount in 1949 described a stapling technique for THE, which provided good results in the correction of angular deformities but was later linked to complications like implant failure and physeal damage (Blount and Clarke 1949, Kanellopoulos et al. 2011, Stevens 2007). In 2007 Stevens introduced a non-locking 2-hole plate (eight-Plate [EP], Orthofix Medical Inc, Lewisville, TX, USA) based on the tension-band principle (Stevens 2007). Even though treatment with the EP seems to have an overall decreased complication rate, screw breakage is still reported rather frequently (Schroerlucke et al. 2009, Burghardt et al. 2010, 2011, Scott 2012, Vogt et al. 2016). This led to the development of a flexible staple (FlexTack [FT], Merete GmbH, Berlin, Germany). Both implants, EP and FT, achieve a tension-band effect through an extra-physeally located fulcrum of correction supposedly leading to reduced compression on the physis, thus decreasing the risk of premature closure of the physis (Vogt et al. 2016). Different from the EP, the FT is a 1-piece implant with a flexible mid-zone that provides dynamic bending under bone growth force.

We examined the physeal response to THE with these 2 tension-band devices and whether there is a histomorphological correlate for the extent of the blockage between FT and EP. Additionally, we sought a possible histomorphological explanation for the excessive unilateral bone growth frequently occurring after implant removal, which might be linked to the incidence of rebound phenomenon.

## Animals and methods

### Study design, animal model, and sample size

**Figure 1.**
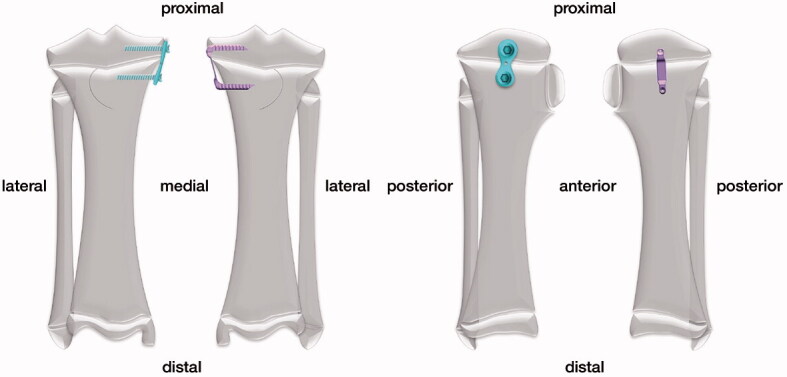
The medial proximal tibia was treated with either FT or EP, while the contralateral tibia received the opposite treatment (in this example right tibia: EP; left tibia: FT).

A randomized paired setup was chosen and a total time period of 14 weeks with histomorphometric analysis after 9 and 14 weeks was set. In order to achieve valid results, the smallest number of animals needed was determined through a statistical power analysis. 12 skeletally immature domestic female pigs were assigned into 2 groups (n = 6). The animal itself served as its control. The average weight of the pigs was 28 (24–33) kg at the time of first surgery. In both groups, each animal received an implantation of either EP or FT on the medial proximal tibia of the right hind leg. The opposite treatment was then performed on the medial proximal tibia of the left hind leg, so both implants were inserted in the same animal (Figure 1). The chosen implant size depended on the size of the proximal tibia.

**Figure 2.**
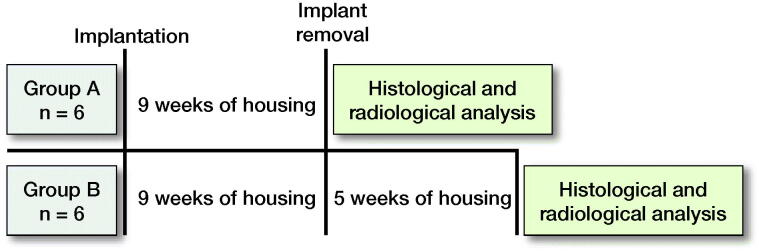
Chronological overview of the two study groups (group A: overall study time 9 weeks; group B: overall study time 14 weeks).

After 9 weeks of housing under the same conditions, implants were removed in both groups. In group A, the histomorphological analysis was performed immediately after implant removal. In group B, animals were housed for another 5 weeks to investigate delayed reactions after physeal release. Thus, the histomorphological analysis in group B was performed after 14 weeks (Figure 2).

### Implants

1 eight-Plate (titanium alloy; 12 mm plate and two 24 mm fully threaded cannulated non-locking screws) and 1 FlexTack (titanium alloy [TiA16V4 ELI]; crossbar 25 mm, epiphysial leg 25 mm, metaphyseal leg 23 mm) were implanted in each pig. The EP is fixated with 2 screws that can be positioned in varying angles and diverge up to 30° during growth. The FT is a 1-piece implant with cannulated legs. Both devices are fixated extraperiosteally to avoid the development of a bone bridge, which might lead to untimely growth arrest. In both implants, the implantation technique is minimally invasive and K-wire guided.

### Surgical technique

General anesthesia was maintained with intravenous propofol (10 mg/kg/h) and fentanyl (25 µg/kg/h) for all surgical interventions on living and ventilated animals. Protocols established by the research group provided the basis for anesthetic techniques, postoperative assessment, and pain care management (Gottliebsen et al. 2013a, Hillebrand et al. 2020).

**Figure 3.**
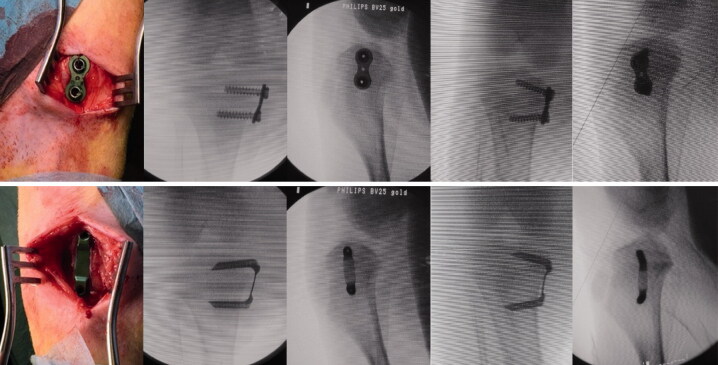
The animals received one centrally placed either EP (upper row) or FT (lower row) on each arrested site. Both implants were inserted in the same animal. Fluoroscopic images show the anteroposterior and lateral plane of the proximal tibia immediately after implantation and before implant removal after nine weeks of bone growth.

A longitudinal incision at the level of the physis was made, which allowed the extra-periosteal insertion of the devices under fluoroscopic control. 1 centrally placed implant (EP or FT) was positioned in the epiphysis and metaphysis. Digital fluoroscopic images were obtained for radiological analysis (Figure 3). After closing the wound, local anesthetics were applied.

After completing the last study procedure, each animal received a lethal dose of pentobarbital (0.5 mL/kg) through intravenous access. Implants were extracted, following the manufacturer’s guidelines. In the case of FT, first the cannulated staple shanks were separated from the bone with a guided U-chisel. Then the extraction instrument was fastened to the FT and removed with the help of a sliding hammer.

After implant removal, both tibiae were harvested and immediately stored at –25 °C until further processing.

### Histological preparation

**Figure 4.**
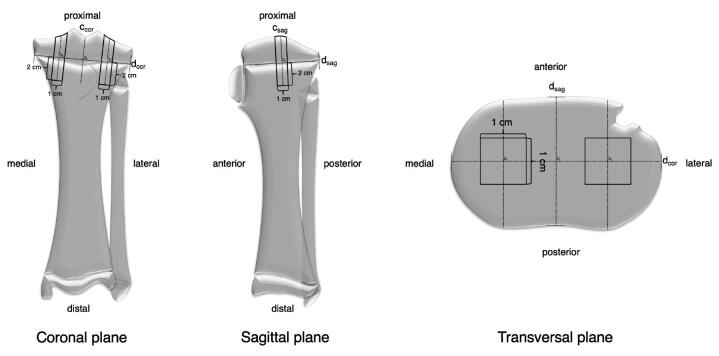
Schematic drawing of standardized bone sample extraction.

All samples were obtained following the same method. 2 lines (dsag and dcor) connecting the outer edges of the tibial plateau were drawn and their point of intersection was determined in the transverse plane. With reference to this point and same distance from each other, 2 square samples (medial and lateral) were taken in a line parallel to the sagittal tibia axis using a sample preparation saw. Each sample was sized 3 cm x 1 cm x 1 cm and consisted of the area of interest with joint cartilage, epiphyseal bone, physeal cartilage, and metaphyseal bone (Figure 4).

**Figure 5.**
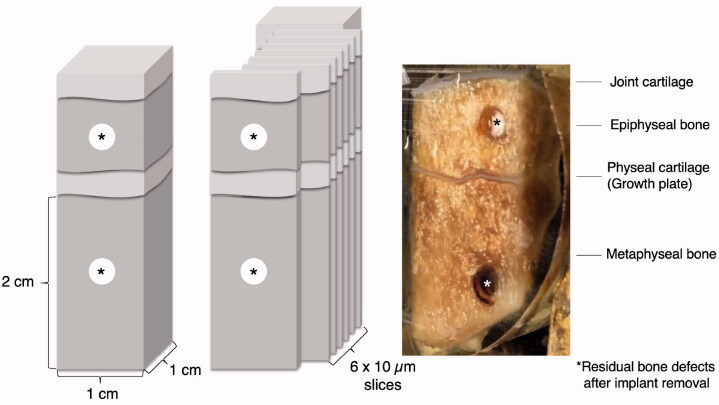
Example of medial bone sample after embedding in MMA.

Before embedding in cold methyl methacrylate, the removed bone samples were dehydrated in graded ethanol (70–100%). From the outer rim of each biopsy, 10 µm-thick coronal sections were cut on a microtome (Polycut E, Reichert-Jung/Leica Microsystems, Wetzlar, Germany). 6 sections were removed from one level and stained with HE (Figure 5).

### Histomorphometric analysis

**Figure 6.**
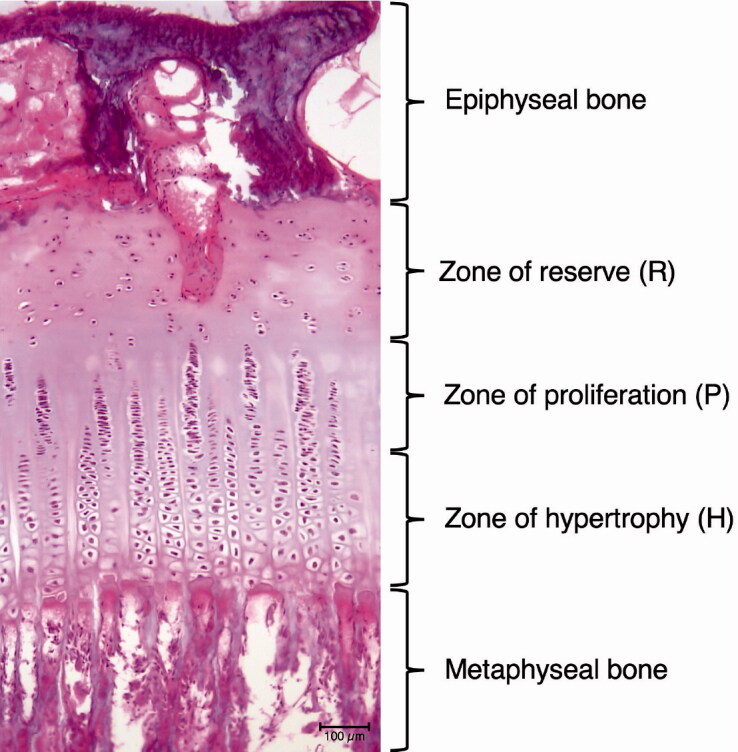
Digital histological images in HE staining at 100x magnification showing zone of reserve (R), proliferative zone (P), and hypertrophy zone (H).

The growth plate is divided into 3 different fractions of chondrocyte layers from epiphysis to diaphysis: zone of reserve (R), zone of proliferation (P), and zone of maturation and hypertrophy (H) (Figure 6) (Nuttall et al. 1999, Byers et al. 2000). Chondrocytes are located in the reverse zone at the epiphyseal end. In the proliferative zone chondrocytes of constant size start aligning in columns, before enlarging in the hypertrophic zone. In addition to these 3 regions, areas with unorganized cartilage tissue (loss of normal columnar appearance of the cell distribution) can be found (Nuttall et al. 1999, Byers et al. 2000, Gottliebsen et al. 2013b).

Normal proportions of the cartilage zones and physiological joint architecture were defined by performing sham surgery on 1 animal in each study group. In these cases the implants were inserted and removed again before closure of the skin.

A microscope (BX50, Olympus, Shinjuku, Japan) was attached to a computer to transmit the microscope field to the computer monitor via video camera. Using software for stereology (newCAST, Visiopharm, Hoersholm, Denmark), on each section, all visible cartilage was outlined as region of interest (ROI) and highlighted. Point counting was used to define the fractions of chondrocyte layers (Gundersen et al. 1988).

### Radiographic evaluation

Anteroposterior (AP) and lateral radiographs of the knee joint were taken immediately before implantation and prior to implant removal after 9 weeks in both group A and B. In group B additionally radiographs were taken after 14 weeks, before the histomorphological samples were harvested.

To investigate changes in the tibial angulation, the medial proximal tibial angle (MPTA) and the articular line-diaphysis angle (ALDA) were analyzed. The MPTA is defined as the medial angle between the mechanical axis of the tibia and the tangent along the articular surface of the tibial plateau (joint line). The ALDA is measured between the anatomical axis of the tibia and the joint line, based on the description of Aykut et al. (2005).

Loss of correction was investigated in group B and defined as the difference in MPTA and ALDA at the time of implant removal (9 weeks after implantation) and 5 weeks after implant removal.

**Figure 7.**
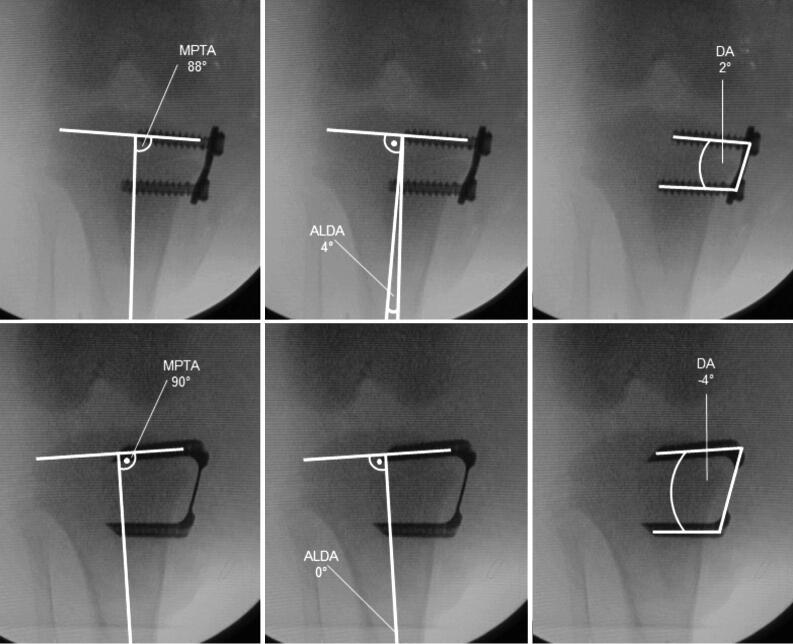
MPTA, ALDA, and DA immediately after implantation of EP (upper row) and FT (lower row).

The effectiveness of the respective implant was determined through the device angle (DA; angle between the epiphyseal and metaphyseal screw or shank, respectively), which was measured on the radiographs taken at implantation and after 9 weeks, prior to implant removal (Figure 7).

### Reproducibility

The reliability of the histomorphometric analysis has previously been tested by the research group estimating the coefficients of variation (CV) for determination of the zonal distribution of the growth plate. The CV values were (very) good for all zones, ranging from 7% to 11% (Gottliebsen et al. 2013b).

The reliability of the radiographic measurements was tested by 2 raters (JS, BV). Rater 1 performed all the measurements just as 56 double-measurements, and rater 2 performed an additional 56 measurements. This correlates with a double-measurement rate of 33.3%. Intraclass correlation coefficients (ICC) were used to determine the inter-rater reliability. A 2-way mixed effects model using absolute agreement definition was used. The estimated ICC values were excellent for all angles, ranging from 0.927, 95% confidence interval (CI) (0.808–0.972) to 0.996 (CI 0.989–0.999), p < 0.001. Regardless of the measured angle, the overall ICC value for all 56 measurements was 0.999 (CI 0.998–0.999), p < 0.001. The analysis was performed with SPSS 25 (IBM Corp, Armonk, NY, USA).

### Power analysis and statistics

EP and FT were implanted on the medial side of the leg. Compared with the lateral, a reduction of the growth plate was expected. A mean difference of 125 units and a standard deviation (SD) of 85 units, a significance level of α = 5% and a test power (1–β) of at least 80% results was assumed. As a result, 6 animals were needed to detect a statistically significant difference by a t-test for paired samples.

Measurements on the same animals were compared using a paired t-test. For the comparisons of individuals between those who were observed for 9 weeks and those who were observed for 14 weeks a 2-sample Welch’s t-test was used. A p-value < 0.05 was considered to be statistically significant. Analysis was performed using R language (R version 3.6.2 [2019-12-12]) (R Core Team 2019).

### Ethics, funding, and potential conflicts of interest

The Danish Animal Research Guidelines and the European Directive 2010/63/EU for animal experiments formed the basis for the experimental protocol (Danish Animal Research Guidelines, European Directive). The Danish Animal Experiment Committee agreed with the research proposal (file number 2015-15-0201-00761).

This study was fully financed by the research funds of the University Hospital of Muenster, Germany. RR is a paid consultant of Merete Medical GmbH. All other authors declare no conflicts of interest.

## Results

All 12 animals tolerated the interventions well and did not show either infections or any abnormalities in eating or movement behavior.

### Histomorphometric results

**Figure 8.**
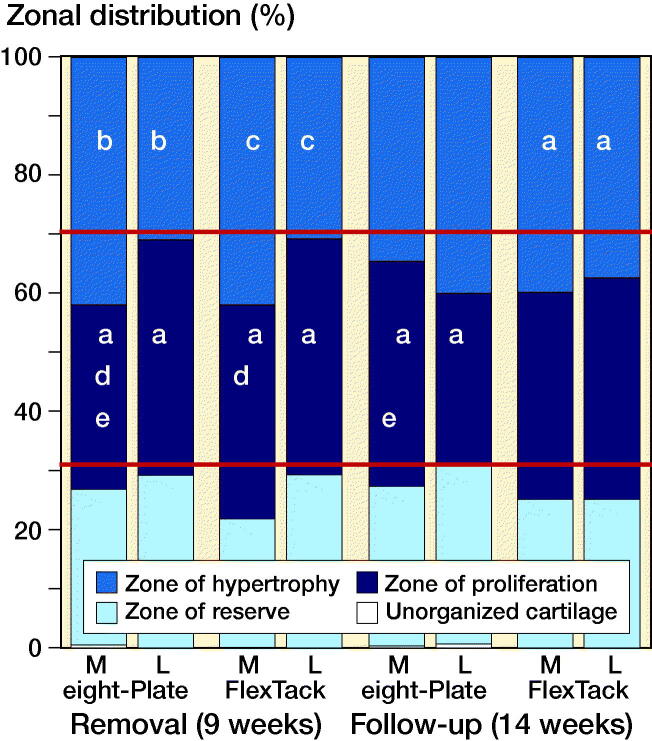
Left side: Zonal distributions after 9 weeks of THE with EP and FT. Right side: Zonal distributions 5 weeks after implant removal. The red horizontal lines show the physiological zone distribution of the porcine growth plate, determined through sham surgery. Significance to the same zone in the same implant (a–c), and between implants (d) in the same period as well as significance to the same zone and implant between the periods (e) is shown: **^a^** p < 0.05, **^b^** p < 0.01, **^c^** p < 0.001, **^d^** p < 0.05, **^e^** p < 0.05.

In comparison with the physiological structure of the growth plate, the zonal distribution of the lateral physis remained unchanged after 9 weeks of THE with either EP or FT. Comparing the medial with the lateral part of the physis after 9 weeks, medially a reduction of the proliferative zone was detected after treatment with EP (p = 0.04), while the reserve zone increased (p = 0.01). THE with FT led to a reduction in both proliferative (p = 0.03) and hypertrophic zone (p < 0.001), while the reserve zone also increased (p < 0.001). THE with EP led to a greater decrease of the proliferative zone on the medial side of the physis than THE with FT (p = 0.02). In total, EP and FT achieved an equal reduction of the proliferative and hypertrophic zone (Figure 8).

5 weeks after removal of the EP, the proliferative zone of the medial physis showed an increase (p = 0.02), while laterally a reduction of the proliferative zone was observed (reversal of distributions). The proportion of the proliferative zone medially was greater than laterally (p = 0.05). The distribution of the hypertrophic zone remained mostly unchanged (Figure 8).

After removal of the FT, the medial physis showed only slight changes of the proliferative zone with increase of the hypertrophic zone, while in the lateral growth plate a marginal reduction of proliferative and hypertrophic zones was observed. The zonal distributions of the proliferative and hypertrophy zone were almost equal medially and laterally, while the proportion of the reserve zone medially was greater (p = 0.04) (Figure 8).

### Radiological results

**Figure 9.**
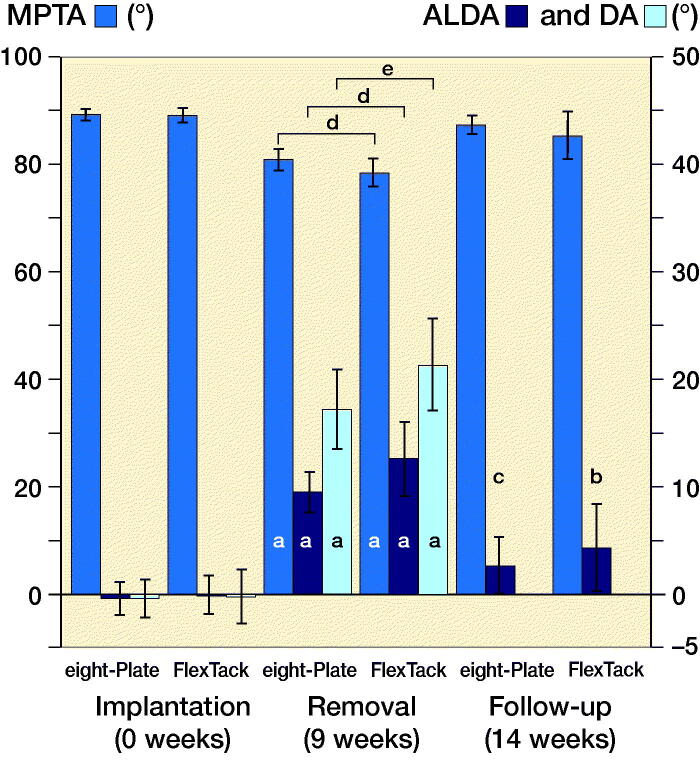
Both EP and FT achieved a significant reduction of MPTA and a significant increase of ALDA and DA after 9 weeks of THE. After 5 weeks both implants showed a significant loss of correction of MPTA and ALDA. The overall reduction of MPTA and increase of ALDA after 14 weeks were not significant. Significance to the same angle in the same implant during implantation (a), removal (b–c), and between implants (d–e) is shown: **^a^**p < 0.001, **^b^**p < 0.01, **^c^**p < 0.001, **^d^**p < 0.01, **^e^**p < 0.001.

**Figure 10.**
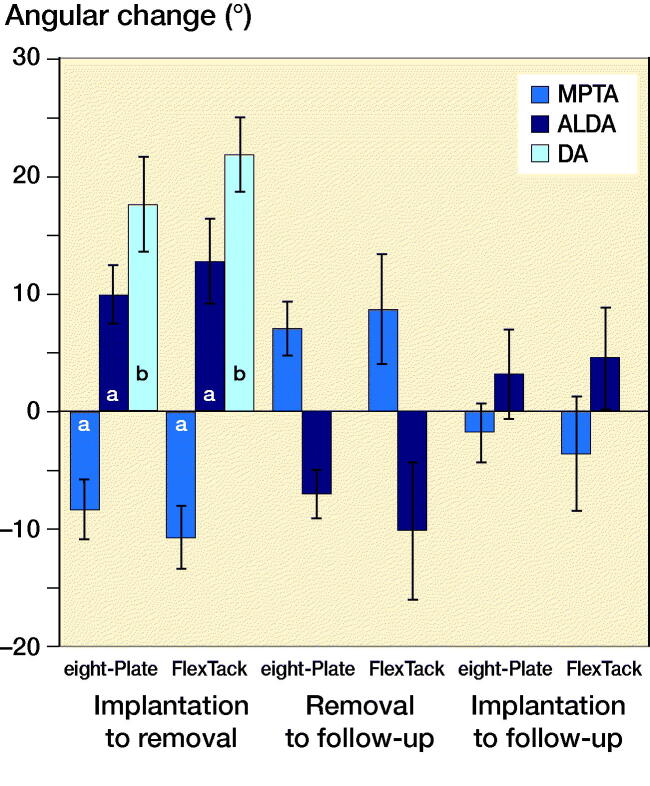
After 9 weeks of THE, the FT achieved a significantly greater decrease of the MPTA and a significantly greater increase of the ALDA and DA. 5 weeks after implant removal, the increase of the MPTA and decrease of the ALDA showed no significant differences between the 2 implants. The overall reduction of MPTA and increase of ALDA at the time of the last follow-up after 14 weeks also proved not to be significantly different between EP and FT. Significance to the same angle in the same period between the implants (a–b) is shown: **^a^**p < 0.05, **^b^**p < 0.001.

**Figure 11.**
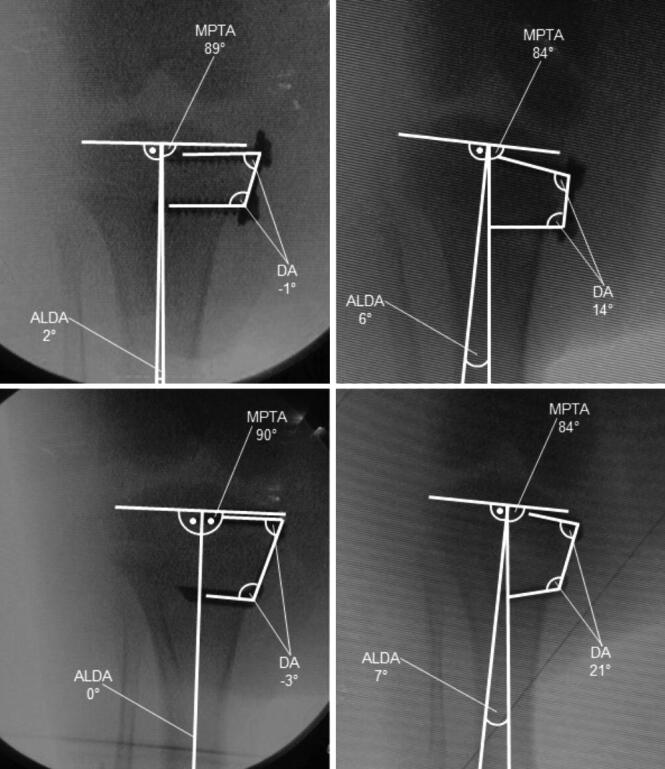
Decrease of the MPTA and increase of ALDA and DA after 9 weeks of THE with EP (upper row) and FT (lower row).

After 9 weeks of THE, both implants, EP and FT, showed a reduction of the MPTA (p < 0.001) (Figure 9). The FT overall achieved a greater decrease than the EP (p = 0.02), with an average reduction of the MPTA of –11° using the FT and –8° using the EP (Figure 10). The ALDA increased after treatment with both EP and FT (p < 0.001) (Figure 9). The FT achieved a greater increase of the ALDA than the EP (p = 0.02), with an increase of the ALDA of an average +13° using the FT and +10° using the EP (Figure 10). EP as well as FT showed an increase of the DA within 9 weeks of THE (p < 0.001) (Figures 9 and 11). The FT showed a greater change of the DA than the EP (p < 0.001), with an average divergence of +22° in the FT and +18° in the EP (Figure 10).

5 weeks after implant removal, both implants showed a loss of correction with a statistically significant increase of the MPTA and a significant decrease of the ALDA (Figure 9). No significant differences in the amount of loss of correction were observed between FT and EP (Figure 10). The average loss of correction of the MPTA was +7° using the EP and +9° using the FT. The average loss of correction of the ALDA was –7° using the EP and –10° using the FT (Figure 10).

At the final follow-up after 14 weeks, the EP showed an overall reduction of the MPTA of on average –2° and an overall increase of the ALDA of on average +3° (Figure 10). The FT showed an overall decrease of the MPTA of on average –4° and an increase of the ALDA of on average +5° (Figure 10). The overall reduction of MPTA and increase of ALDA after 14 weeks was not significant in either implant (Figures 9 and 10).

## Discussion

Even though the EP has become a widely popular device for growth guiding treatment, hardware failure—in particular breakage of the metaphyseal screw—is a rather frequent complication (Schroerlucke et al. 2009, Burghardt et al. 2010, Burghardt et al. 2011, Scott 2012, Vogt et al. 2016). A possible explanation for screw failure might be the maximal divergence of the screws in the correction of severe angular deformities. Under sustained growth the plate consequently bends in the other direction, which might lead to blockage of the screws within the plate and subsequent screw breakage. Furthermore, Burghardt et al. stated that the EP has a delayed onset of correction in comparison with fixed angle staples (Burghardt et al. 2011). As the screws of the EP initially show a mechanical slackness within the 2-hole plate, correction is induced only after a certain degree of angulation and sufficient bone purchase is achieved (Burghardt et al. 2011). Eltayeby et al. (2019), on the other hand, did not detect a significant correlation between the initial screw angle and the average rate of correction; they stated that a wider initial screw angle did not result in a faster correction rate. Vogt et al. (2016) reported an earlier correction start and faster correction speed of the FT in comparison with the EP. They attributed this to the 1-piece design of the FT, which presumably leads to immediate correction commencement and prevents breakage of the implant.

In our study, neither EP nor FT showed implant failures. The radiological evaluation revealed an adequate alteration of the angular axes with both devices. However, the FT achieved a faster correction than the EP after 9 weeks of THE. The higher mean DA in THE with FT was related to greater angular correction. In group A, both implants showed a statistically significant reduction in the proportion of the proliferative zone of the arrested medial physis in comparison with the lateral physis and with the physiological zonal distribution. The FT also achieved a significant reduction of the hypertrophy zone. The reduction in the proportions of hypertrophic and proliferative zones of the medial physis seemed to be more pronounced after THE with FT, which might be a histomorphological correlation to the radiological findings.

Deformity recurrence occurring after implant removal before skeletal maturity is a common problem in growth modulation treatment (Stevens et al. 1999, Burghardt and Herzenberg 2010, Farr et al. 2018, Leveille et al. 2019). The likelihood of relapse of the deformity is reportedly higher if THE is performed at a young age, in the correction of secondary axis deviations or an angular axis deviation over 20°, and in the case of a fast correction rate (Stevens and Klatt 2008, Park et al. 2016, Farr et al. 2018, Leveille et al. 2019). Nonetheless, the occurrence of rebound phenomenon shows a very variable incidence, as the physeal response after release of the epiphysiodesis is hardly predictable (Aykut et al. 2005, Leveille et al. 2019). Widening of the formerly arrested part of the physis after release of the physeal compression has been described by several authors, and has been linked to accelerated unilateral growth with consecutive relapse of the initial deformity (Gottliebsen et al. 2013b), Corominas-Frances et al. 2015, Ding et al. 2018).

In our study, 5 weeks after implant removal restoration of the physiological cartilaginous zone distributions appeared to be slightly prolonged after THE with FT. This might indicate a minor risk for the occurrence of a rebound phenomenon. In comparison, after THE with EP the return to the physiological proportions of the medial physis appeared to take place more rapidly, with increase in the proportion of the proliferative cartilage in the formerly arrested physeal zone. The lateral, previously not blocked side of the growth plate showed a paradox reduction of the proliferative zone. This might be a possible histological correlate for the occurrence of rebound phenomenon. Radiologically, however, a rebound—or rather return to the physiological limb alignment—was observed in all cases and was similar between EP and FT.

This study has several limitations. Apart from the small cohort and short period of observation, the results of THE in a porcine model may differ from those of THE in skeletally immature children. Furthermore, as previously described by Corominas-Frances et al. (2015), THE was performed in tibiae initially presenting physiological axes, in which an angular deformity was produced. In our study, what was interpreted as a rebound deformity may in fact have been bone remodeling to regain physiological limb alignment. Additionally, it is unclear whether the fact that the contralateral leg of the same pig has been used as a control may have influenced the results.

## Conclusion

Both tension band devices investigated achieved THE in the porcine model. A substantial inhibition of the growth plate was obtained in all cases without causing physeal damage. Neither EP nor FT showed any implant-related complications. The radiographic evaluation revealed a substantial change of angular axes and DA in THE with both implants, while the correction appeared to be faster with FT. In all cases a rebound was observed 5 weeks after implant removal. Histomorphologically, the growth inhibition of the medial physis seemed to be slightly more pronounced after treatment with FT, while showing prolonged restoration of the physiological zonal distributions after implant removal. On the other hand, after removal of the EP a paradox cartilaginous reaction was observed. These observations could be possible histomorphological correlates for the rebound phenomenon.

## References

[CIT0001] Aykut U S, Yazici M, Kandemir U, et al. The effect of temporary hemiepiphyseal stapling on the growth plate: a radiologic and immunohistochemical study in rabbits. J Pediatr Orthop 2005; 25(3): 336–41. 15832150 10.1097/01.bpo.0000152906.23669.d8

[CIT0002] Blount W P, Clarke G R. Control of bone growth by epiphyseal stapling: a preliminary report. J Bone Joint Surg Am 1949; 31A(3): 464–78. 18153890

[CIT0003] Burghardt R D, Herzenberg J E. Temporary hemiepiphysiodesis with the eight-Plate for angular deformities: mid-term results. J Orthop Sci 2010; 15(5): 699–704. 20953936 10.1007/s00776-010-1514-9

[CIT0004] Burghardt R D, Kanellopoulos A D, Herzenberg J E. Hemiepiphyseal arrest in a porcine model. J Pediatr Orthop 2011; 31(4): e25-9. 21572268 10.1097/BPO.0b013e31821a5d04

[CIT0005] Burghardt R D, Specht S C, Herzenberg J E. Mechanical failures of eight-plate-guided growth system for temporary hemiepiphysiodesis. J Pediatr Orthop 2010; 30(6): 594–7. 20733426 10.1097/BPO.0b013e3181e4f591

[CIT0006] Byers S, Moore A J, Byard R W, et al. Quantitative histomorphometric analysis of the human growth plate from birth to adolescence. Bone 2000; 27(4): 495–501. 11033444 10.1016/s8756-3282(00)00357-4

[CIT0007] Corominas-Frances L, Sanpera I, Saus-Sarrias C, et al. Rebound growth after hemiepiphysiodesis: an animal-based experimental study of incidence and chronology. Bone Joint J 2015; 97-B(6): 862–8. 26033070 10.1302/0301-620X.97B6.34822

[CIT0008] Danish Animal Research Guidelines. Available from: https://www.retsinformation.dk/Forms/R0710.aspx?id=145380.

[CIT0009] Ding J, He J, Zhang Z Q, et al. Effect of hemiepiphysiodesis on the growth plate: the histopathological changes and mechanism exploration of recurrence in mini pig model. Biomed Res Int 2018; 2018: 6348171. 30687754 10.1155/2018/6348171 PMC6330884

[CIT0010] Eltayeby H H, Iobst C A, Herzenberg J E. Hemiepiphysiodesis using tension band plates: does the initial screw angle influence the rate of correction? J Child Orthop 2019; 13(1): 62–6. 30838077 10.1302/1863-2548.13.180086 PMC6376435

[CIT0011] European Directive. 2010/63/EU for animal experiments. Available from: http://eara.eu/en/animal–research/eu-animal-research-law-directive-2010-63/.2010/63/EU for animal experiments.

[CIT0012] Farr S, Alrabai H M, Meizer E, et al. Rebound of frontal plane malalignment after tension band plating. J Pediatr Orthop 2018; 38(7): 365–9. 27574955 10.1097/BPO.0000000000000846

[CIT0013] Gottliebsen M, Moller-Madsen B, Stodkilde-Jorgensen H, et al. Controlled longitudinal bone growth by temporary tension band plating: an experimental study. Bone Joint J 2013a; 95-B(6): 855–60. 23723285 10.1302/0301-620X.95B6.29327

[CIT0014] Gottliebsen M, Rahbek O, Poulsen H D, et al. Similar growth plate morphology in stapling and tension band plating hemiepiphysiodesis: a porcine experimental histomorphometric study. J Orthop Res 2013b; 31(4): 574–9. 23192490 10.1002/jor.22276

[CIT0015] Gundersen H J, Bagger P, Bendtsen T F, et al. The new stereological tools: dissector, fractionator, nucleator and point sampled intercepts and their use in pathological research and diagnosis. APMIS 1988; 96(10): 857–81. 3056461 10.1111/j.1699-0463.1988.tb00954.x

[CIT0016] Hillebrand H, Sattelberger J, Gosheger G, et al. Comparison of temporary epiphysiodesis with RigidTacks and Blount-Staples in a porcine animal model using magnetic resonance imaging. J Orthop Res 2020; 38(5): 946–53. 31743488 10.1002/jor.24532

[CIT0017] Kanellopoulos A D, Mavrogenis A F, Dovris D, et al. Temporary hemiepiphysiodesis with Blount staples and eight-Plates in pigs. Orthopedics 2011; 34(4). 10.3928/01477447-20110228-05 21469635

[CIT0018] Leveille L A, Razi O, Johnston C E. Rebound deformity after growth modulation in patients with coronal plane angular deformities about the knee: who gets it and how much? J Pediatr Orthop 2019; 39(7): 353–8. 31305378 10.1097/BPO.0000000000000935

[CIT0019] Nuttall J D, Brumfield L K, Fazzalari N L, et al. Histomorphometric analysis of the tibial growth plate in a feline model of mucopolysaccharidosis type VI. Calcif Tissue Int 1999; 65(1): 47–52. 10369733 10.1007/s002239900656

[CIT0020] Park S S, Kang S, Kim J Y. Prediction of rebound phenomenon after removal of hemiepiphyseal staples in patients with idiopathic genu valgum deformity. Bone Joint J 2016; 98-B(9): 1270–5. 27587531 10.1302/0301-620X.98B9.37260

[CIT0021] R Core Team. R: A language and environment for statistical computing. R Foundation for Statistical Computing, Vienna, Austria; 2019. Available from: https://www.R-project.org/.

[CIT0022] Schroerlucke S, Bertrand S, Clapp J, et al. Failure of Orthofix eight-Plate for the treatment of Blount disease. J Pediatr Orthop 2009; 29(1): 57–60. 19098648 10.1097/BPO.0b013e3181919b54

[CIT0023] Scott A C. Treatment of infantile Blount disease with lateral tension band plating. J Pediatr Orthop 2012; 32(1): 29–34. 22173384 10.1097/BPO.0b013e31823db034

[CIT0024] Stevens P M. Guided growth for angular correction: a preliminary series using a tension band plate. J Pediatr Orthop 2007; 27(3): 253–9. 17414005 10.1097/BPO.0b013e31803433a1

[CIT0025] Stevens P M, Klatt J B. Guided growth for pathological physes: radiographic improvement during realignment. J Pediatr Orthop 2008; 28(6): 632–9. 18724199 10.1097/BPO.0b013e3181841fda

[CIT0026] Stevens P M, Maguire M, Dales M D, et al. Physeal stapling for idiopathic genu valgum. J Pediatr Orthop 1999; 19(5): 645–9. 10488868

[CIT0027] Vogt B, Kleine-König M-T, Gosheger G, et al. FlexTack^TM^ and RigidTack^TM^: new devices for correction of angular deformities and leg length discrepancies by temporary epiphysiodesis. J Child Orthop 2016; 10: 17–8.

